# Mining microsatellites in the peach genome: development of new long-core SSR markers for genetic analyses in five *Prunus* species

**DOI:** 10.1186/s40064-015-1098-0

**Published:** 2015-07-10

**Authors:** Maria Teresa Dettori, Sabrina Micali, Jessica Giovinazzi, Simone Scalabrin, Ignazio Verde, Guido Cipriani

**Affiliations:** Consiglio per la Ricerca in Agricoltura e l’analisi dell’economia agraria, Centro di Ricerca per la Frutticoltura, Rome, Italy; IGA Technology Services, Udine, Italy; Dipartimento di Scienze Agrarie e Ambientali, University of Udine, Udine, Italy

**Keywords:** *P. armeniaca*, *P avium*, *P. persica*, *P. salicina*, *P. dulcis*, Fingerprinting

## Abstract

A wide inventory of molecular markers is nowadays available for individual fingerprinting. Microsatellites, or simple sequence repeats (SSRs), play a relevant role due to their relatively ease of use, their abundance in the plant genomes, and their co-dominant nature, together with the availability of primer sequences in many important agricultural crops. Microsatellites with long-core motifs are more easily scored and were adopted long ago in human genetics but they were developed only in few crops, and *Prunus* species are not among them. In the present work the peach whole-genome sequence was used to select 216 SSRs containing long-core motifs with tri-, tetra- and penta-nucleotide repeats. Microsatellite primer pairs were designed and tested for polymorphism in the five diploid *Prunus* species of economic relevance (almond, apricot, Japanese plum, peach and sweet cherry). A set of 26 microsatellite markers covering all the eight chromosomes, was also selected and used in the molecular characterization, population genetics and structure analyses of a representative sample of the five diploid *Prunus* species, assessing their transportability and effectiveness. The combined probability of identity between two random individuals for the whole set of 26 SSRs was quite low, ranging from 2.30 × 10^−7^ in peach to 9.48 × 10^−10^ in almond, confirming the usefulness of the proposed set for fingerprinting analyses in *Prunus* species.

**Electronic supplementary material:**

The online version of this article (doi:10.1186/s40064-015-1098-0) contains supplementary material, which is available to authorized users.

## Background

The *Prunus* genus includes several diploid species of economic relevance. Comparative mapping studies showed that the genomes of the diploid *Prunus* species are essentially colinear and syntenic (Dettori et al. 2001; Dirlewanger et al. 2004; Verde et al. 2005; Dondini et al. 2007; Jung et al. 2009) and DNA fingerprinting of accessions belonging to these species consistently revealed a high transportability of molecular markers (Cipriani et al. 1999; Dirlewanger et al. 2002; Vendramin et al. 2007). Fingerprinting based on molecular markers is a popular tool for studies of population genetics and diversity, including the resolution of synonymy/homonymy controversies, the protection of plant breeders’ rights, paternity and kinship analyses.

SSR markers (simple sequence repeats), or microsatellites, consist of tandemly repeated DNA sequences with a core unit of 1–6 base pairs (bp). They offer a number of positive features for the genetic profiling of individuals including wide distribution in plant genomes, prevalent single-locus tagging in diploid species, multi-allelic co-dominant patterns, simple use and availability of several primer sequences in many important agricultural crops (Schlötterer 2004). The high variability of microsatellites is mainly due to a different number of repeats in the region of the repeated motif but also to short insertion/deletion events (Decroocq et al. 2003).

In humans and animals long nucleotide repeats, namely tetra- and penta- motifs, were adopted because neighbor alleles are more easily separated from each other (Hammond et al. 1994; Ruitberg et al. 2001; Butler et al. 2004; Butler 2006; Hellmann et al. 2006). Moreover, di-nucleotide SSRs, even though frequent in eukaryotic genomes, suffer from the presence of ghost bands (stuttering), which make the interpretation of electropherograms and the allele call less reliable.

The first SSRs developed by plant scientists were mainly di-nucleotide repeats, which are the most abundant in plant genomes. The isolation procedure was costly, microsatellites were isolated from SSR-enriched libraries with the aim of producing a high number of potentially useful markers for mapping purposes. The availability of whole-genome sequences offers the opportunity to mine the genomes and retrieve thousands of different kind of markers including single nucleotide polymorphisms (SNPs), structural variants and microsatellites.

SNPs are widely used for the generation of saturated genetic maps due to the availability of high-throughput automated genotyping platforms (Gunderson 2009). High-throughput SNP tools have been recently developed in *Prunus* species using an Illumina platform (Peace et al. 2012; Verde et al. 2012) and have been used to genotype cultivars and accessions to perform large scale genetic analyses (Micheletti et al. 2012). However, mapping technologies using SNP markers are still rather expensive and not applicable in every laboratory.

Due to their relative abundance in the genome and simple relatively low cost detection, microsatellites are still preferable in population genetics and fingerprinting studies with a low or moderate number of markers. As the regions flanking the repeated motif are in many cases highly conserved, microsatellite markers are easily amplified by PCR in many different accessions and close species. Long-core repeats microsatellites have been developed in a few tree species: grape (Cipriani et al. 2008, 2010), *Eucalyptus* (Faria et al. 2011) and olive (De la Rosa et al. 2013).

The availability of the peach genome sequence (Verde et al. 2013) has allowed the scanning of the whole genome with the aim of retrieving microsatellites to be used for molecular analyses in peach and in its closely related species belonging to the *Prunus* genus.

The aim of this study was to find a universal set of polymorphic tri-, tetra- or penta-nucleotide SSRs distributed in the eight chromosomes for the following diploid *Prunus* species: peach (*P. persica*), almond (*P. dulcis*), apricot (*P. armeniaca*), Japanese plum (*P. salicina*), sweet cherry (*P. avium).* These SSRs were also required to preferably be single locus and to have common amplification parameters.

## Methods

### Retrieving microsatellites from the peach genome sequence

Penta-, tetra- tri- and di-nucleotide core simple sequence repeats with a minimum length of 12 bp were retrieved from the peach whole-genome sequence (Peach v1.0; Verde et al. 2013) available at the Phytozome web site (http://www.phytozome.net/peach; Goodstein et al. 2012). A modified version of the software Sputnik (Abajian 1994) with the default parameters was used. Microsatellite sequences were scanned in each one of the eight pseudomolecules of the genome assembly (scaffolds 1–8), representing the eight *Prunus* chromosomes and containing up to 96% of the total peach sequence (Verde et al. 2013).

The final goal was to select a useful set of well-distributed markers, at least two for each chromosome. To improve polymorphism detection rate we chose to consider only microsatellites longer than 19 bp. Within each pseudochromosome, SSRs were chosen giving preference to those located towards the ends to ensure marker independence. Each microsatellite-containing sequence was aligned against the peach genome dataset through the BLASTn algorithm available at the Phytozome website to check adjacent regions; microsatellites falling within or close to repetitive regions were excluded. Primers were designed for the selected SSR loci using Primer 3 software (http://bioinfo.ut.ee/primer3-0.4.0/primer3/) and were subjected to BLASTn analysis against the Peach v1.0 to discard those targeting multiple loci. The parameters for primer design were as follows: amplicon size 150–300 bp, primer size 18–24 bp, primer melting temperature of 56–58°C with an optimum at 57°C, max self-complementarity 3 or 4 (3 preferred) and max 3′ self-complementarity 1 or 2 (1 preferred).

### Preliminary PCR primer testing

Preliminary PCR amplifications were performed for each designed marker in a panel of the five *Prunus* diploid species. In total 24 accessions were used as follows: eight peaches, eight cherries, three Japanese plums, three almonds and two apricots. The final number of microsatellites was thus achieved in a stepwise procedure of selection and testing until a minimum number of efficient primers for a given chromosome was reached.

Young leaves were collected from plants at the germplasm collection field of the CRA Centro di Ricerca per la Frutticoltura—Rome (Centro Nazionale Germoplasma Frutticolo—CNGF), frozen in liquid nitrogen and stored at −80°C until freeze dried. DNA was extracted using the Qiagen DNeasy Plant Mini Kit, following the manufacturer’s instructions. PCR reactions were carried out in a volume of 10 μL with a final concentration of 200 μM of each dNTP and 0.1 μM of each primer, 10 ng genomic DNA, and 0.5U of Platinum^®^Taq DNA Polymerase (Invitrogen). An Applied Biosystem Verity thermal cycler was used with the following thermal profile: one cycle at 94°C for 5 min, followed by 10 touch down cycles at 94°C for 30 s, the primer specific Tm°C—0.5°C/cycle for 45 s, 72°C for 60 s, followed by 25 cycles at 94°C for 30 s, Ta°C for 45 s, 72°C for 60 s, and a final step of 30 min at 72°C. The annealing temperature of each primer pair is reported in Additional file 1: Table S1. Amplicons were separated in a 3% MetaPhor™ Agarose (Lonza) gel in TBE 1 × buffer and scored, after GelRed™ staining, for the presence of bands.

### PCR primer testing

Primers polymorphic in at least three different species were re-tested on a total of 18 cultivars for each species (Table 1), chosen on the basis of previous fingerprinting studies with the aim of maximizing genetic diversity. DNA samples were amplified as explained above, but using WellRED forward primers (0.075 µM) labeled with D2-PA, D3-PA and D4-PA (Sigma-Aldrich) fluorescent dyes. D3-PA- and D4-PA-labeled PCR products were diluted 1:5 and 1:9, respectively, in ddH2O, while D2-PA-labeled amplicons were left undiluted. One microliter from each of the three PCR reactions was analyzed in multiplex, by adding 0.5 µl of CEQ DNA size Standard kit 400 (Beckman Coulter) and 36.5 µl of CEQ sample loading solution for a total of 40 µl. Amplicons were separated by capillary electrophoresis, performed on a CEQ8000 DNA Analysis System (Beckman Coulter).

**Table 1:** The 90 accessions belonging to the five *Prunus* species used to test the long-core repeat primer pairs selected in the peach genome

Species	Cultivar/accession	Pedigree	Species	Cultivar/accession	Pedigree
Almond	Cavaliera	–	Peach	Alexandra	Noblesse op
	Cristomorto	–		Babygold 8	Pl35201 × Ambergem
	Desmayo Largueta	–		Catherina	NJC95 × D42-13 W
	Desmayo Rojo	–		Duchessa d’Este	(Mayflower × Amsden) op
	Ferraduel	Cristomorto × Aï		Elberta	Chinese Cling op or Chinese Cling × Early Crawford
	Ferragnes	Cristomorto × Aï		Fantasia	GoldKing × Red King op
	Genco	–		Ferganensis	–
	Glorieta	Primorskii × Cristomorto		Gialla di Verona	–
	Malaguena	–		Maruja	–
	Marcona	–		Maycrest	Springcrest mutant
	Masbovera	Primoskii × Cristomorto		Oro A	Diamante op
	Moncayo	Tardive de la Verdiere × Tuono		Quetta	–
	Nonpareil	–		Redhaven	Halehaven × Kalhaven
	Pizzuta d’Avola	–		Royal Moon	–
	Primorskii	–		Sahua Hong Pantao	–
	Retsou	–		Shenzhou Mitao	–
	Texas	Ferragnès × Tuono		Snow Queen	–
	Tuono	–		Yumyeong	Yamato-Wase × Nunome Wase
Apricot	Bergeron	–	Sweet cherry	Adriana	ISF 123 × Mora di Cazzano
	Bulida	–		Bianca di Aritzo	–
	Castelbrite	–		Burlat	Seedling (unknown origin)
	Comédie	Bergeron × Rouge de Roussillon		Casanova	–
	Currots	–		Durone nero II	–
	Dany	–		Ferrovia spur	Ferrovia mutant
	Helena	–		Isabella	Starking Hardy Giant × Stella
	Lady Rose	–		Kasthanka	–
	Magyar Kajszi	Hungarian Best		Kavics	Germersdorfi orias 92 × Budakalasz
	Ninfa	Ouardi × Tyrinthos		Lambert	–
	Palstein	–		Lapins	Van × Stella
	Pinkot	–		Linda	–
	S. Castrese	–		Pagliaccio	–
	Stella	–		Rainier	Bing × Van
	Goldrich	SunGlo × Perfection		Schneider Späte Knorpel	–
	Tardive de Bordaneil	–		Stella	Lambert × Jhon Innes
	Tyrinthos	–		Sunburst	Van × Stella
	Vicario	–		Van	Imperatrice Eugenie op
Japanese plum	Angeleno	Queen Anne op			
	Black Diamond	–			
	Black Star	–			
	Friar	Gaviota × Nubiana			
	Golden Kiss	–			
	Laetitia	–			
	Larry Ann	–			
	Methley	–			
	Obilnaya	–			
	Oishi Nakate	–			
	Queen Rosa	Santa Rosa × Queen Anne			
	Sangue di Drago	–			
	Santa Rosa	–			
	September Giant	–			
	T.C. Sun	–			
	Tracy Sun	–			
	Weeping Santa Rosa	–			
	Yummy Beaut	–			

### Data analysis

SSRs were analyzed with the fragment analysis tool of the software CEQ Genetic Analysis System v 8.0 (Beckman Coulter). Genotypes showing a single peak at a given locus were recorded as homozygous. Single locus allelic data were used for population genetic parameters and stratification estimates. Cervus 3.0.6 (Kalinowski et al. 2007) was used for the calculation of allele frequency, observed and expected heterozygosity (Ho and He, respectively), the polymorphic information content (PIC), which measures the marker locus informativeness in relation to the expected heterozygosity, and the probability of identity, defined as the probability of two unrelated individuals sharing the same genetic profile by chance (NE-I). Frequencies of null alleles were calculated using the IIM (Individual Inbreeding Model) Bayesian approach implemented in the INEST software (Chybicki and Burczyk 2009) setting the cycles to 500,000 and the thinning parameter to 8,000. In order to define the best model fitting the data, the deviance information criterion (DIC) tool, available in the advanced 2.0v of the software, was computed both under the complete set of parameters (nfb model—simultaneous presence of null alleles, inbreeding and random amplification failure) and without inbreeding (nb model). A permutation test was also performed to estimate heterozygosity excess based on the inbreeding coefficient estimates (F_IS_ = 1 − Ho/He) and a 95% confidence interval of the null distribution of F was obtained after 1,000,000 random permutations of all alleles among genotypes.

The ability of the microsatellite set to reveal population structure was evaluated using the model-based clustering method implemented in the software Structure 2.3.4 (Pritchard et al. 2000). For each species analyses were performed for K ranging from one to nine for ten independent replications under the admixture model with no prior population information. Tests were carried out applying a burn-in period of 75,000 followed by 200,000 Monte Carlo Markov chain (MCMC) iterations. The true number of K was chosen applying the Evanno method (Evanno et al. 2005) implemented in the online software Harvester (Earl and vonHoldt 2012); the software CLUMPP v. 1.1.2 (Jakobsson and Rosenberg 2007) was used, employing the full search algorithm, to find the optimal alignment of the ten independent replicate cluster analyses and to compute the mean membership coefficient matrix (Q-matrix). This matrix was entered into DISTRUCT v1.1 (Rosenberg 2004) to obtain an ordered graphical display of the population structure.

Relations among entries were analyzed using the software DARwin v 6.0 (Perrier and Jacquemoud-Collet 2006) scoring the data as presence/absence to include multilocus alleles. The dissimilarity matrix between accessions was calculated using the Dice index and the UPGMA tree was constructed using the hierarchical clustering method.

The newly developed markers were also compared with long core microsatellites already published to check for locus uniqueness.

## Results and discussion

### Selection of microsatellites

The primary aim of this study was to produce a set of long-core repeat SSR markers suitable for genetic analysis and genotyping in five different species of *Prunus* (almond, apricot, Japanese plum, peach and sweet cherry).

A total of 63,145 microsatellites carrying di-, tri-, tetra- and penta-nucleotide repeats were recovered from the peach genome sequence assembly (Peach v1.0;Verde et al. 2013). Di-nucleotide microsatellites were the most frequent in the peach genome (48.2%) followed by penta- (22.8%), tetra- (14.7%) and tri-nucleotide (14.3%) core motif microsatellites (Table 2). Microsatellites were arbitrarily divided into two classes: class I consists of perfect core repeats with more than 19 bp and class II consists of 12–19 bp long repeats (Table 2). The two class sizes were chosen following the classification already adopted in rice (Temnykh et al. 2001). The number of perfect microsatellites assigned to the two classes was 32,038 (50.7%) and 31,107 (49.3%), respectively. Within Class I long-core motifs, penta-nucleotide microsatellites were confirmed to be the most represented in the peach genome (13%), followed by those with repeats three (10%) and four (8%) nucleotides long. The relative abundance of the three types of long-core microsatellites found in our study is different from that described by Shi et al. (2013) scanning the same *Prunus persica* genome assembly dataset with a different computer program. However, this is not surprising as in several species (human, *Saccharomyces cerevisiae*, *Neurospora crassa* and *Drosophila melanogaster*) the distribution of microsatellites found within the genome was greatly variable in relation to different parameter settings and to the algorithm used for microsatellite detection (Leclercq et al. 2007).

**Table 2:** Repeats retrieved from the peach genome sequence

SSR Type	Class^a^	N. loci	Total length (bp)	Reference genome (%)^b^	SSR length	
					Min	Max
Dinucleotide	I	21,964	740 830	0.33	20	306
	II	8,503	137 806	0.06	14	18
	*Tot*	*30,467*	*878,636*	*0.39*	*14*	*306*
Trinucleotide	I	3,198	87,378	0.04	21	390
	II	5,819	93,459	0.04	15	18
	*Tot*	*9,017*	*180,837*	*0.08*	*15*	*390*
Tetranucleotide	I	2,566	57,996	0.03	20	88
	II	6,708	96,236	0.04	12	16
	*Tot*	*9,274*	*154,232*	*0.07*	*12*	*88*
Pentanucleotide	I	4,310	97,620	0.04	20	200
	II	10,077	151,155	0.07	15	15
	*Tot*	*14,387*	*248,775*	*0.11*	*14*	*200*
*Tot*	*Class I*	*32,038*	*983,824*	*0.43*	*20*	*390*
	*Class II*	*31,107*	*478,656*	*0.21*	*12*	*18*
	*Tot*	*63,145*	*1,462,480*	*0.64*	*12*	*390*

^a^SSRs longer than 19 bp were assigned to class I, those in the range between 12 and 19 bp were assigned to class II.

^b^based on 227,252,106 bp in peach v1.0.

Microsatellite distribution was homogeneous across the genome, with an average of one microsatellite every 3.5 kb (Class I and Class II) and minimal differences among the pseudomolecules.

### Preliminary testing of the primers pairs

A total of 222 long-core repeat SSRs from the Peach v1.0 database was tested (Additional file 1: Table S1). Out of these selected sequences, 74 contained tri-nucleotide motifs, 67 tetra-nucleotide motifs, and 81 penta-nucleotide motifs (Additional file 1: Table S1).The largest number of microsatellites was selected from the peach chromosome 4 (ten, nine, and 20 tri-, tetra-, and penta-nucleotides, respectively), and the lowest number from chromosome 2 (six, five, and six tri-, tetra-, and penta-nucleotides, respectively). However, this distribution is not representative of the relative chromosome lengths or of the actual distribution of the microsatellites across the genome, but is likely due to a bias in the stepwise selection procedure.

Peach samples were amplified at 216 SSR loci; six primer pairs did not yield an amplicon in any of the five species, peach included, and were therefore excluded from subsequent analyses.

A survey of the microsatellites already available in peach, revealed that the vast majority has a dinucleotide motif. None of our 216 microsatellites targets the same locus as the long-core SSRs previously published.

One hundred eighty-eight primer pairs (87.0%) did amplify all the five species; the overall SSR cross-transportability value obtained in this study is quite high and is in agreement with those previously observed in the genus by Dirlewanger et al. (2002), and Vendramin et al. (2007), 75.6 and 95%, respectively. The latter and highest value was found in a set of SSRs developed from the transcriptome of peach fruit. Twenty-eight primers did not yield amplification in at least one species: four failed in two species, two in four species and 22 in one species (15 in sweet cherry). Of the 28 primers not amplifying in at least one species, as much as 21 failed in sweet cherry, which is the more phylogenetically distant from peach (Bortiri et al. 2006). As expected, all primers gave a product in peach, the species from which these SSRs had been selected. A search in the list of predicted protein-coding genes from the peach genome sequence (v1.0) detected 68 markers (31.5%) out of 216 in genic regions; the Peach v1.0 ID for the SSRs containing genes is reported in Additional file 1: Table S1. Differences in the rate of transportability across *Prunus* were observed between genic and intergenic SSRs; five (7.4%) genic SSRs did not amplify in one of the species analyzed while 23 (15.5%) intergenic SSRs did not amplify in at least one species (17 in one species, four in two species and two in four species).

The number of markers polymorphic in at least one species was 153 (70.8%) and the rate of polymorphism of the three types of SSRs was 75.0% in tri-nucleotides, 63.1% in tetra-nucleotides, 73.4% in penta-nucleotides. Ninety-seven primer pairs gave polymorphic patterns in almond (46.6%), 83 in Japanese plum (40.9%), 50 in apricot (24.6%), 45 in peach (21.0%) and 37 in sweet cherry (19.5%; Table 3). Only 21.0% of the SSRs were polymorphic in peach, the species from which the sequences containing the microsatellite regions had been selected. The lower level of variability of peach compared to the other four species found in the present work is well known (Byrne 1990; Mnejja et al. 2010) and is the result of many factors. Peach is in fact, the only self-compatible species of this work and self-pollination, leading to homozygosity, is predominant (Miller et al. 1989; Hegedüs et al. 2006). Moreover, it has undergone severe bottlenecks during domestication and diversification (Verde et al. 2013) and modern peach cultivars were established from a very narrow genetic pool (Scorza et al. 1985; Aranzana et al. 2010). The higher rate of polymorphism found in almond compared to the other species is expected considering that almond is an outcrossing self-incompatible species phylogenetically more strictly related to peach (Bortiri et al. 2006). The rate of polymorphism in almond, apricot and Japanese plum is likely to be underestimated due the lower number of samples analyzed respect to peach and cherry. Differences in polymorphism rates were observed between genic and intragenic markers: SSRs polymorphic in at least one species were 45 (66.2%) genic and 108 (73.0%) intragenic, while those polymorphic in all the species were 2 (2.9%) and 11 (7.4%), respectively. Considering the single species (Table 3) almond was the most polymorphic one having a rate of polymorphism of 42.4% for genic and 48.6% for intergenic SSRs. Cherry was the least polymorphic, with a rate of 14.3 and 22.0% in genic and intergenic regions, respectively.

**Table 3:** Distribution of 216 SSR markers between genic and intergenic regions and relative polymorphisms in the five *Prunus* species

	Almond (n = 3)	Apricot (n = 2)	Japanese plum (n = 3)	Peach (n = 8)	Sweet cherry (n = 8)
tot scorable SSRs	208	204	204	214	190
tot polymorphic SSRs	97	50	83	45	37
% polymorphic SSRs	46.6	24.6	40.9	21.0	19.5
tot scorable genic SSRs	66	65	63	68	63
tot polymorphic genic SSRs	28	16	19	12	9
% polymorphic genic SSR	42.4	24.6	30.2	17.6	14.3
tot scorable intergenic SSRs	142	139	141	146	127
tot polymorphic intergenic SSRs	69	34	64	33	28
% polymorphic intergenic SSRs	48.6	24.5	45.4	22.6	22.0

*n* number of accessions used for testing.

Twenty-six of these 222 primer pairs met the criteria needed to enter the next step of analysis, the remaining being discarded due to one or more drawbacks such as weak amplification, unreadable multi-peak profiles, monomorphic profile and/or amplification failure in more than two species.

### Evaluation of SSR profiles and polymorphism

The primer pair characteristics and the diversity parameters of the 26 long-core SSRs in each of the five *Prunus* species are listed in Tables 4 and 5, respectively. Thirteen primer pairs identified polymorphisms in all the species (Table 5), ten in four species and three in three species, respectively. At least one polymorphic marker was found for each chromosome in all the species.

**Table 4:** Characteristics of the 26 long-core repeat primer pairs selected for fingerprinting of the five *Prunus* species

SSR ID	Pseudochrom.	T_A_	(SSR motif)_n_	Primer Forward		Primer Reverse	
				Sequence	Peach v1.0 start	Sequence	Peach v1.0 start
RPPG1-017	1	56	(AGCTT)_5_	GCTCATCAAAACTCTCAACCA	2,785,626	CCCTTTCTTCAATCCCATC	2,785,848
RPPG1-026	1	51	(GAT)_7_	CTTCTGGCACTCTTCCATTT	4,980,754	GTTCCCAAGTTTTCCTCTCA	4,980,991
RPPG1-032	1	53	(CTT)_7_	ATGGCAGAGAGCACAACAA	22,022,341	TTGAGAGGTAACAGCGAGAA	22,022,564
RPPG1-037	1	53	(AGC)_7_	GTCTCTGATCCAAGCCAACT	42,186,590	ACGCTGCCATTGTTTCTATT	42,186,831
RPPG1-041	1	56	(ATT)_7_	TGTTGTAATGGATGGTGTCTTC	44,374,756	CTTGGTCTTGGTTTCATTCA	44,374,983
RPPG2-011	2	53	(ATTT)_5_	TTTACAGGTGCCTCAACAAA	3,728,089	GTACAGCCGATGGAGAGAAA	3,728,287
RPPG2-022	2	53	(CTGT)_6_	CTGCTGCGTCTGATGATG	26,576,155	ACAGGACAGGACCACTTTCT	26,576,364
RPPG3-026	3	53	(CTGT)_6_	AGAACGCTATTCCCCTGTAA	3,151,168	TCATCCTCTCCAAATGTCAA	3,151,412
RPPG4-059	4	56	(ACTGG)_6_	GACGGCTGTTTATTTGCATT	138,756	TGCATTTGTGATCTCGTTTC	138,937
RPPG4-067	4	58	(GGTTT)_4_	AGAAGGGAGGGTGAGAGAAG	3,564,871	CACGAAGGAAGAAACGAAGT	3,565,136
RPPG4-077	4	57	(AATT)_5_	CCTCGTCTTCAGTCTTTTCTG	18,611,275	CTGTCCCTTCTGTGTTCCTAA	18,611,433
RPPG4-084	4	58	(ATTT)_5_	TCCTCAAAAGTTACCCCAAG	26,137,915	CTTGCTGTGGAAGAAGAACC	26,138,190
RPPG4-091	4	49	(CTTTT)_6_	GGAGGGTAGAGAACAGAGCA	27,055,301	CGGAAGATGTGATTGTGAGA	27,055,542
RPPG5-018	5	56	(ATT)_8_	GCATGAAATTGACCCATACA	5,331,336	TAATTGCTTTGGGGAGGAC	5,331,523
RPPG5-022	5	58	(ATC)_11_	CTTGTGAACTGGCATCTGTC	8,805,836	AGTTGTATGGGCATGTTGTG	8,806,134
RPPG5-023	5	56	(ATT)_7_	TTGTTTGCACTAGGCTTTGA	16,625,324	TTCTTCTTGCATGTCCTTGA	16,625,517
RPPG5-025	5	53	(CCCTT)_5_	GTGTCTCCTCCTCAAAGCAA	16,792,568	TACGGCAACCAAGAACATC	16,792,866
RPPG5-030	5	53	(AATT)_5_	AAGGCAAGGAATTGGGTAGT	18,027,410	TGGTTTGTCGTAAGAGTCCA	18,027,575
RPPG6-009	6	53	(GTTTT)_4_	GGGCTTGGCTGATAAAATAA	1,068,427	TGGTAAAATAGAAGAGCGAGAAG	1,068,608
RPPG6-032	6	53	(ATCGC)_5_	TCCTATGGCAAAAACAAAATC	26,949,411	TGAAGAGATGGAGTGGAAGAG	26,949,563
RPPG6-033	6	56	(CTGT)_6_	CATTATCAAACCACGACCAA	27,071,911	AAAGCTCAACAGCGACTTCT	27,072,026
RPPG7-015	7	58	(ATTT)_6_	TCTTGGTGGTGGTGAAGTAA	2,533,650	GAGAGATGGAGGAGGCTGA	2,533,925
RPPG7-026	7	53	(ACATT)_4_	TTTGGTGAGTGGGCTCTATT	18,786,038	CTATCGTTCGCTGGTCTTCT	18,786,203
RPPG7-032	7	53	(AGG)_7_	AAGGGAGGAGGATTGTGAA	22,275,889	TGGTAGACGGGTAGATGTTG	22,276,079
RPPG8-007	8	53	(GGT)_7_	ACCACCACCTCTTCCAATC	86,262	ACCTCAAAGTGTCCCAGAAA	86,469
RPPG8-028	8	58	(AACCC)_6_	AAGGAGCCGACATCAGAAC	20,410,671	TGACCAGAAGCCAAATACATC	20,410,876

*T*_*A*_ annealing temperature, *Peach v1.0 start* location in the peach genome sequence.

**Table 5:** Population statistics of 26 peach-derived long-core repeat SSR markers developed for five *Prunus* species

SSR ID	Pseudo chrom.	N. bases in repeat	N. loci^a^	N. alleles^b^	Alleles range	N. amplified samples^c^	Ho	He	PIC	NE-I	F_IS_	f_null_	N. amplified species
Almond													
RPPG1-017	1	5	1	3	177–188	18	0.444	0.427	0.349	0.408	−0.040	0.0001	5
RPPG1-026	1	3	1	2	239–242	18	0.111	0.108	0.099	0.807	−0.028	0.0038	4
RPPG1-032	1	3	1	1	221	18	–	–	–	–	–	–	4
RPPG1-037	1	3	1	3	230–242	18	0.833	0.684	0.591	0.186	−0.218	0.0011	4
RPPG1-041	1	3	1	8	238–255	18	0.833	0.768	0.712	0.099	−0.085	0.0087	5
RPPG2-011	2	4	>1	12	203–277	18	–	–	–	–	–	–	5
RPPG2-022	2	4	>1	10	208–239	18	–	–	–	–	–	–	5
RPPG3-026	3	4	>1	4	241–253	18	–	–	–	–	–	–	5
RPPG4-059	4	5	1	2	132–161	18	0.056	0.056	0.053	0.896	0	0.0198	3
RPPG4-067	4	5	1	4	261–275	18	0.611	0.662	0.596	0.174	0.077	0.0020	3
RPPG4-077	4	4	1	4	131–151	18	0.611	0.703	0.617	0.167	0.131	0.0159	5
RPPG4-084	4	4	1	5	271–297	18	0.556	0.638	0.545	0.219	0.129	0.0159	5
RPPG4-091	4	5	1	3	245–249	18	0.056	0.500	0.424	0.327	0.888*	0.1512	5
RPPG5-018	5	3	1	8	160–197	18	0.778	0.765	0.707	0.102	−0.017	0.0122	4
RPPG5-022	5	3	>1	7	271–301	18	–	–	–	–	–	–	3
RPPG5-023	5	3	1	2	192–193	18	0.667	0.489	0.362	0.388	−0.364	0.0111	4
RPPG5-025	5	5	>1	5	301–316	18	–	–	–	–	–	–	4
RPPG5-030	5	4	1	2	163–167	18	0.167	0.157	0.141	0.729	−0.064	0.0123	5
RPPG6-009	6	5	1	3	181–191	18	0.333	0.494	0.388	0.362	0.326	0.0335	4
RPPG6-032	6	5	>1	3	141–151	18	–	–	–	–	–	–	4
RPPG6-033	6	4	>1	9	93–130	18	–	–	–	–	–	–	5
RPPG7-015	7	4	>1	8	290–332	18	–	–	–	–	–	–	4
RPPG7-026	7	5	1	2	152–161	18	0.444	0.457	0.346	0.407	0.028	0.0086	5
RPPG7-032	7	3	1	4	186–201	18	0.611	0.575	0.488	0.265	−0.063	0.0025	5
RPPG8-007	8	3	1	3	189–214	18	0.389	0.414	0.363	0.396	0.060	0.0045	5
RPPG8-028	8	5	1	4	192–215	18	0.722	0.660	0.583	0.187	−0.094	0.0020	4
*Mean* ^*d*^	–	–	–	*3.6*	–	–	*0.474*	*0.503*	*0.433*	*9.48* *×* *10* ^−*10*^	–	–	–
Apricot													
RPPG1-017	1	5	1	3	165–169	18	0.500	0.579	0.473	0.281	0.136	0.0080	5
RPPG1-026	1	3	1	2	223–235	18	0.389	0.500	0.368	0.382	0.222	0.0151	4
RPPG1-032	1	3	1	2	227–234	18	0.056	0.056	0.053	0.896	0	0.0131	4
RPPG1-037	1	3	1	5	228–251	18	0.389	0.694	0.633	0.148	0.439*	0.0171	4
RPPG1-041	1	3	1	3	212–214	18	0.056	0.652	0.558	0.21	0.914*	0.1480	5
RPPG2-011	2	4	>1	4	194–218	18	–	–	–	–	–	–	5
RPPG2-022	2	4	>1	7	215–241	18	–	–	–	–	–	–	5
RPPG3-026	3	4	1	2	239–249	18	0.389	0.322	0.264	0.521	−0.208	0.0043	5
RPPG4-059	4	5	1	1	132	18	–	–	–	–	–	–	3
RPPG4-067	4	5	1	1	259	18	–	–	–	–	–	–	3
RPPG4-077	4	4	1	4	136–151	18	0.222	0.257	0.237	0.575	0.136	0.0061	5
RPPG4-084	4	4	1	2	276–285	18	0.333	0.413	0.321	0.439	0.194	0.0068	5
RPPG4-091	4	5	1	2	217–228	18	0.111	0.108	0.099	0.807	−0.028	0.0128	5
RPPG5-018	5	3	1	4	177–197	15	0.333	0.582	0.465	0.288	0.428	0.1536	4
RPPG5-022	5	3	1	3	271–299	18	0.444	0.560	0.445	0.307	0.207	0.0112	3
RPPG5-023	5	3	1	3	181–206	18	0.444	0.541	0.450	0.301	0.179	0.0101	4
RPPG5-025	5	5	1	2	275–283	18	0.056	0.056	0.053	0.896	0	0.0292	4
RPPG5-030	5	4	1	2	154–167	18	0.056	0.056	0.053	0.896	0	0.0210	5
RPPG6-009	6	5	1	1	191	18	–	–	–	–	–	–	4
RPPG6-032	6	5	1	2	151–157	18	0.278	0.246	0.211	0.607	−0.130	0.0048	4
RPPG6-033	6	4	1	6	101–119	18	0.389	0.670	0.609	0.163	0.419*	0.0331	5
RPPG7-015	7	4	1	4	304–322	17	0.471	0.606	0.513	0.245	0.223	0.0768	4
RPPG7-026	7	5	1	2	161–170	18	0.111	0.108	0.099	0.807	−0.028	0.0129	5
RPPG7-032	7	3	1	2	186–189	18	0.333	0.286	0.239	0.560	−0.164	0.0068	5
RPPG8-007	8	3	1	2	184–193	18	0.111	0.108	0.099	0.807	−0.028	0.0172	5
RPPG8-028	8	5	>1	8	192–215	17	–	–	–	–	–	–	4
*Mean* ^*d*^	–	–	–	*2.9*	–	–	*0.274*	*0.370*	*0.312*	*6.00* *×* *10* ^−*8*^	–	–	–
Japanese plum													
RPPG1-017	1	5	1	5	172–184	18	0.833	0.659	0.593	0.177	−0.264	0.0033	5
RPPG1-026	1	3	1	2	230–235	18	0.056	0.157	0.141	0.729	0.643	0.0401	4
RPPG1-032	1	3	1	2	225–231	18	0.611	0.475	0.355	0.397	−0.286	0.0122	4
RPPG1-037	1	3	1	2	234–237	18	0.167	0.157	0.141	0.729	−0.064	0.0117	4
RPPG1-041	1	3	1	5	219–247	18	0.722	0.616	0.518	0.242	−0.172	0.0053	5
RPPG2-011	2	4	>1	9	196–232	18	–	–	–	–	–	–	5
RPPG2-022	2	4	>1	4	195–265	18	–	–	–	–	–	–	5
RPPG3-026	3	4	1	8	255–355	18	0.889	0.878	0.836	0.039	−0.013	0.0091	5
RPPG4-059	4	5	1	3	155–178	18	0.389	0.338	0.300	0.480	−0.151	0.0148	3
RPPG4-067	4	5	1	2	265–270	18	0.167	0.157	0.141	0.729	−0.064	0.0184	3
RPPG4-077	4	4	1	3	125–136	18	0.056	0.252	0.226	0.589	0.778*	0.0975	5
RPPG4-084	4	4	1	3	285–330	18	0.278	0.675	0.582	0.192	0.588*	0.1501	5
RPPG4-091	4	5	1	4	226–258	18	0.778	0.605	0.522	0.236	−0.286	0.0044	5
RPPG5-018	5	3	1	7	160–197	15	0.467	0.717	0.643	0.144	0.349*	0.2096	4
RPPG5-022	5	3	>1	6	252–299	18	–	–	–	–	–	–	3
RPPG5-023	5	3	1	2	181–184	18	0.167	0.157	0.141	0.729	−0.064	0.0091	4
RPPG5-025	5	5	1	3	277–285	18	0.667	0.589	0.479	0.277	−0.132	0.0060	4
RPPG5-030	5	4	1	3	154–167	18	0.611	0.475	0.378	0.374	−0.286	0.0103	5
RPPG6-009	6	5	1	2	186–191	18	0.500	0.386	0.305	0.461	−0.295	0.0094	4
RPPG6-032	6	5	1	2	147–152	18	0.444	0.356	0.286	0.488	−0.247	0.0101	4
RPPG6-033	6	4	>1	5	101–119	18	–	–	–	–	–	–	5
RPPG7-015	7	4	>1	9	230–316	18	–	–	–	–	–	–	4
RPPG7–026	7	5	1	4	143–161	18	0.556	0.713	0.638	0.149	0.220	0.0252	5
RPPG7-032	7	3	1	2	186–189	18	0.167	0.246	0.211	0.607	0.321	0.0351	5
RPPG8-007	8	3	1	2	180–194	18	0.056	0.056	0.053	0.896	0	0.0160	5
RPPG8-028	8	5	>1	8	189–224	18	–	–	–	–	–	–	4
*Mean* ^*d*^	–	–	–	*3.3*	–	–	*0.429*	*0.433*	*0.374*	*6.50* *×* *10* ^−*10*^	–	–	–
Peach													
RPPG1-017	1	5	1	4	205–227	18	0.389	0.490	0.437	0.314	0.206	0.0051	5
RPPG1-026	1	3	1	1	239	18	–	–	–	–	–	–	4
RPPG1-032	1	3	1	2	221–225	18	0.056	0.056	0.053	0.896	0	0.0050	4
RPPG1-037	1	3	1	1	242	18	–	–	–	–	–	–	4
RPPG1-041	1	3	1	2	223–231	18	0.333	0.457	0.346	0.407	0.271	0.0050	5
RPPG2-011	2	4	1	2	203–226	18	0.500	0.386	0.305	0.461	−0.295	0.0014	5
RPPG2-022	2	4	1	2	209–214	18	0.222	0.356	0.286	0.488	0.376	0.0069	5
RPPG3-026	3	4	1	2	245–249	18	0.500	0.513	0.374	0.376	0.025	0.0008	5
RPPG4-059	4	5	1	3	176–187	18	0.444	0.589	0.479	0.277	0.246	0.0055	3
RPPG4-067	4	5	1	1	271	18	–	–	–	–	–	–	3
RPPG4-077	4	4	1	2	151–160	18	0.333	0.514	0.375	0.375	0.352	0.0107	5
RPPG4-084	4	4	1	2	279–281	18	0.389	0.513	0.374	0.376	0.242	0.0020	5
RPPG4-091	4	5	1	2	239–245	18	0.444	0.508	0.372	0.378	0.126	0.0049	5
RPPG5-018	5	3	1	2	177–190	18	0.444	0.489	0.362	0.388	0.092	0.0003	4
RPPG5-022	5	3	1	1	301	18	–	–	–	–	–	–	3
RPPG5-023	5	3	1	2	192–196	18	0.333	0.286	0.239	0.560	−0.164	0.0022	4
RPPG5-025	5	5	1	1	301	18	–	–	–	–	–	–	4
RPPG5-030	5	4	1	2	167–169	18	0.056	0.056	0.053	0.896	0.000	0.0044	5
RPPG6-009	6	5	1	2	181–196	18	0.111	0.108	0.099	0.807	−0.028	0.0062	4
RPPG6-032	6	5	1	1	151	18	–	–	–	–	–	–	4
RPPG6-033	6	4	1	2	116–118	18	0.500	0.475	0.355	0.397	−0.053	0.0024	5
RPPG7-015	7	4	1	4	275–303	18	0.611	0.537	0.463	0.287	−0.138	0.0025	4
RPPG7-026	7	5	1	3	157–175	18	0.278	0.541	0.414	0.337	0.486*	0.0044	5
RPPG7-032	7	3	1	2	193–196	18	0.111	0.108	0.099	0.807	−0.028	0.0014	5
RPPG8-007	8	3	1	3	205–214	18	0.056	0.160	0.149	0.719	0.650	0.0082	5
RPPG8-028	8	5	1	2	207–212	18	0.167	0.386	0.305	0.461	0.567	0.0029	4
*Mean* ^*d*^	–	–	–	*2.4*	–	–	*0.314*	*0.376*	*0.297*	*2.30* *×* *10* ^−*7*^	–	–	–
Sweet cherry													
RPPG1-017	1	5	1	3	176–196	17	0.059	0.169	0.157	0.705	0.651	0.1703	5
RPPG1-026	1	3	1	2	274–298	18	0.389	0.437	0.334	0.421	0.110	0.0117	4
RPPG1-032	1	3	1	3	225–231	18	0.833	0.589	0.479	0.277	−0.414*	0.0058	4
RPPG1-037	1	3	1	5	237–265	18	0.444	0.635	0.543	0.220	0.301	0.0209	4
RPPG1-041	1	3	1	6	222–237	17	0.706	0.742	0.682	0.116	0.049	0.0172	5
RPPG2-011	2	4	>1	4	185–224	18	–	–	–	–	–	–	5
RPPG2-022	2	4	1	5	221–234	18	0.889	0.778	0.713	0.102	−0.143	0.0026	5
RPPG3-026	3	4	1	2	239–249	18	0.111	0.108	0.099	0.807	−0.028	0.0183	5
RPPG4-059	4	5	1	1	132	18	–	–	–	–	–	–	3
RPPG4-067	4	5	1	2	260–265	18	0.056	0.056	0.053	0.896	0	0.0204	3
RPPG4-077	4	4	1	3	141–148	18	0.444	0.514	0.449	0.301	0.136	0.0167	5
RPPG4-084	4	4	1	2	287–290	17	0.529	0.515	0.375	0.375	−0.027	0.0312	5
RPPG4-091	4	5	1	4	245–290	18	0.778	0.717	0.649	0.140	−0.085	0.0011	5
RPPG5-018	5	3	1	1	179	18	–	–	–	–	–	–	4
RPPG5-022	5	3	1	1	295	17	–	–	–	–	–	–	3
RPPG5-023	5	3	1	0	–	0	–	–	–	–	–	–	4
RPPG5-025	5	5	1	4	275–289	18	0.722	0.694	0.607	0.173	−0.040	0.0096	4
RPPG5-030	5	4	1	3	163–167	18	0.389	0.522	0.404	0.346	0.255	0.0224	5
RPPG6-009	6	5	1	2	197–207	18	0.556	0.413	0.321	0.439	−0.346	0.0067	4
RPPG6-032	6	5	1	2	147–152	18	0.056	0.056	0.053	0.896	0	0.0125	4
RPPG6-033	6	4	1	3	95–105	17	0.824	0.540	0.414	0.336	−0.526*	0.0123	5
RPPG7-015	7	4	1	0	–	0	–	–	–	–	–	–	4
RPPG7-026	7	5	1	2	175–178	18	0.167	0.157	0.141	0.729	−0.064	0.0052	5
RPPG7-032	7	3	1	3	192–201	18	0.556	0.538	0.412	0.338	−0.033	0.0077	5
RPPG8-007	8	3	1	3	196–208	18	0.389	0.332	0.285	0.496	−0.172	0.0061	5
RPPG8-028	8	5	1	1	201	18	–	–	–	–	–	–	4
*Mean* ^*d*^	–	–	–	*3.1*	–	–	*0.468*	*0.448*	*0.378*	*2.39* *×* *10* ^−*9*^	–	–	–

*Ho* observed heterozygosity, *He* expected heterozygosity, *PIC* polymorphic information content, *NE-I* Probability of Identity, *F*_*IS*_ inbreeding coefficient, *fnull* frequencies of null alleles.

* value significantly different from zero at α < 0.05.

^a^Multilocus markers (N. loci >1) were not used to calculate population parameters.

^b^for multilocus SSRs the number reported refers to the number of fragments.

^c^There are no “missing data” in this matrix. If the number of amplified samples is <18 the sample amplification did not gave a product in at least two repetitions and was thus considered as a null allele (see the main text for details).

^d^Means calculated exluding monomorfic and multilocus SSRs; For NE-I the combined probability of identity is reported.

Nine SSRs detected more than one locus in at least one species. However, even if patterns were more complex due to the higher number of peaks, all primer pairs resulted in a high quality scoring. All the markers confirmed to target single loci in peach, whereas a multi locus pattern was more frequent in the other diploid *Prunus* species: eight were found in almond, six in Japanese plum, three in apricot and one in sweet cherry. As reported by Verde et al. (2013) *Prunus* did not undergo recent whole genome duplication. However, a segmental duplication has been described in peach in MADS-box (Bielenberg et al. 2008) and in MYB transcription factors (Zhou et al. 2014). Duplicated SSR loci have also been often described in *Prunus*, as may be highlighted in the different linkage maps obtained in the last decades (Dirlewanger et al. 2004; Verde et al. 2005; Dondini et al. 2007). All the 26 primer pairs were polymorphic in Japanese plum, 25 in almond, 23 in apricot, 20 in cherry and 20 in peach, respectively.

The number of alleles per locus varied depending on the species, with the highest, equal to 8, found in almond and Japanese plum. The average number of alleles per species ranged from 2.4 (in peach) to 3.6 (in almond).The highest mean observed heterozygosity within the five species was found in almond (0.474) closely followed by sweet cherry (0.468) and Japanese plum (0.429); in peach and apricot it was 0.314 and 0.274, respectively. Heterozygosity values reported in literature for each of these species show wide range of variation depending on many factors such as the number and choice of accessions, the SSR set used and the electrophoretic system chosen for fragment separation. We obtain here values (Table 5) that are generally lower than those reported in literature. However all the previous works used shorter motif repeats (mostly di-nuclotides), which are known to be more variable than long core repeats (Chakraborty et al. 1997; Vigouroux et al. 2002).

Inbreeding coefficients (Table 5) generally displayed values slightly different from zero. Based on the permutation test, F_IS_ values were found significantly different from zero at α < 0.05 in a few loci, distributed across all the five species: one single locus in peach and almond, two loci in cherry and three loci each in plum and apricot. Departures from Hardy–Weinberg equilibrium (HWE) had in all cases a positive sign, revealing an excess of homozygotes, with the exception of cherry, where both the markers (RPPG1-032 and RPPG6-033) displayed an excess of heterozygotes. The excess of homozygotes in a population departing from HWE could indicate the presence of null alleles, which is not easily verifiable without direct observation such as in segregation or parentage analyses. To account for the presence of null alleles avoiding biases due to inbreeding, estimation of null allele frequency was performed under the model with the lowest DIC value, as estimated with the INEST software. The nbf model, simultaneously accounting for inbreeding and null alleles, was found to better fit data for all the species with the exception of Japanese plum.

The frequencies of null alleles (f_null_) are listed in Table 5. Among loci displaying a significant excess in homozygotes, the lowest value was found, as expected, in peach (0.004) and the highest in Japanese plum with marker RPPG5-018 (0.210). This marker displayed three unamplified samples both in Japanese plum and apricot, thus supporting the presence of a null allele, already hypothesized after repeating the amplifications. In apricot, two (‘Bergeron’ and ‘Comedie’) of the three cultivars independently scored as homozygous for a null allele are known to have a parent-offspring relationship (Table 1). In a few cases the IIM estimate of null allele presence is consistent with homozygote–homozygote mismatches in known parent-offspring relationships. For some accessions parental relationships were available from literature (Table 1): in apricot, the cv Ninfa is known to be an offspring of ‘Tyrinthos’ (Table 1). The genotyping results with our set of markers are all compatible with this pedigree with the exception of the RPPG1-041 marker. For this locus, showing a homozygous pattern with the Tyrinthos 213 allele, admitting the presence of a null allele could meet the genealogy of the cultivar. The same happens with marker RPPG4-091 in almond for cv Tuono (parent) and Moncayo (offspring).

The known parental relationships (Table 1) were also used to assess the effectiveness of the marker set for parentage analyses. In sweet cherry pedigree information could be fully confirmed for one cultivar, being both parents present in our genotyped materials, and for three further varieties it was compatible with the genetic profile of the single parent present in our panel. The parental relationship of ‘Van’ as offspring of ‘Rainier’ was found inconsistent in two different loci (RPPG5-030 and RPPG4-091). In almond, six cultivars could be assessed (Table 1) but only two pedigrees could be confirmed: ‘Ferragnes’ and ‘Ferraduel’, sharing ‘Cristomorto’ as parent. The four unmatching results could be explained by mislabeling in one of the many steps involved in the collection setup, and further analysis should be performed to confirm or discard the pedigrees.

The highest PIC index was found in plum (0.836), while the highest average value was found in almond (0.433). The locus RPPG1-041 was the most informative, with the highest average PIC value in the five *Prunus* species (0.563), and the locus RPPG6-032 was the least informative (average PIC = 0.183). The efficiency of the peach-derived long-core repeat markers was different in the five species tested. Fourteen primer pairs showed a PIC value higher than 0.300 in almond, a threshold under which markers are considered scarcely polymorphic (Botstein et al. 1980). Likewise, 11 primer pairs showed a PIC value higher than 0.300 in Japanese plum, 13 in sweet cherry, 13 in peach and 10 in apricot (Table 5). Further multilocus highly variable primer pairs were found that could be useful in fingerprinting and paternity tests: eight in almond (3–12 different fragments), six in plum (4–9 fragments), one in cherry. The combined probability of identity (combined NE-I) between two random individuals for the whole set of 26 SSRs was quite low ranging from 2.30 × 10^−7^ in peach to 9.48 × 10^−10^ in almond, confirming the usefulness of the proposed set for fingerprinting analyses in *Prunus* species.

**Figure 1:**
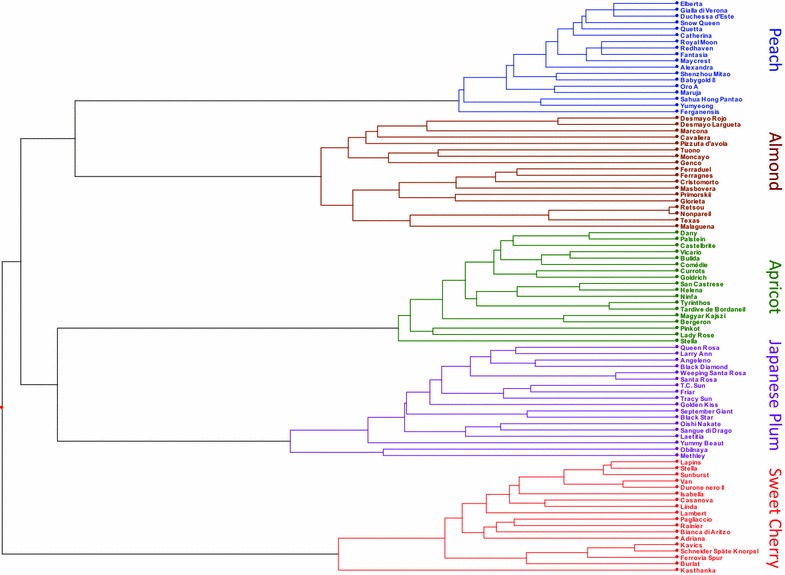
Tree obtained from the analysis of 90 accessions belonging to five *Prunus* species. The tree was constructed with the UPGMA method using the complete tri-, tetra-, and penta-nucleotide data set.

All the eighteen cultivars of each of the five *Prunus* species could be genetically identified with the set of long-core repeat SSR markers (Figure 1). Relationships among the species shown in the dendrogram were in agreement with the classification proposed by Bortiri et al. (2006) with peach and almond closely linked and belonging to the *Amygdalus* subgenus, *Prunus armeniaca* and *Prunus salicina* more distant and belonging to the *Prunus* sensu lato; all the four species belonging to a single clade. *Prunus avium,* belonging to a different clade is classified as *Cerasus* subgenus. Genetic distances displayed in the tree were obtained by using the full dataset, including multilocus markers, while population stratification results were obtained by using only single locus markers. Both these datasets gave a similar representation of the relationships inside each species.

**Figure 2:**
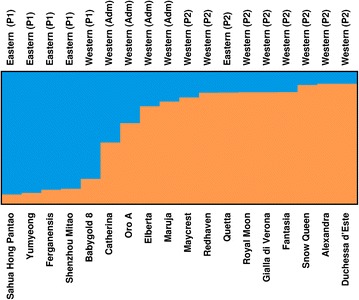
Population stratification of 18 peach accessions. Above the figure it is reported the location where the accessions were collected or developed and, in *brackets*, the two subgroups (K = 2) to which they were assigned by STRUCTURE. *Adm* admixed, *P1* subgroup 1 (*light grey*), *P2* subgroup 2 (*dark grey*).

The developed set of markers was able to reveal population structure in all the five species analyzed; an accession was declared as part of a subpopulation when its membership coefficient was higher than 0.8. In peach, two subpopulations (K = 2) were estimated, which can be ascribed to the eastern (P1, five accessions) and western (P2, nine accessions) germplasm as already observed by Micheletti et al. (2012) and Li et al. (2013). The oriental group included four known oriental accessions (‘Sahua Hong Pantao’, ‘Yumyeong’, ‘Ferganensis’, ‘Shenzhou Mitao’) and ‘Babygold 8’; the latter is a western cultivar obtained in the USA but in accordance to our results, it is reported to have not less than 75% of Chinese blood (Okie 1998). The nine accessions included in P2 are all of well-known western origin, with the exception of Quetta, an old nectarine cultivar collected in 1906 in India, already reported to cluster with western germplasm by Verde et al. (2013, supplementary information). The obtained peach population structure is represented in Figure 2. In the above mentioned works (Micheletti et al. 2012; Li et al. 2013), carried out with a larger number of plant materials and markers, the best population stratification estimate was at K = 3 as the western subpopulation resulted further divided into modern and traditional accessions. In cherry, three subpopulations were observed comprising 15 accessions. One of the subpopulations had three samples in common with the modern cultivars subpopulation identified by Mariette et al. (2010). Two further shared cultivars, belonging to the landrace group in the results of Mariette et al. (2010), were admixed in our work. In apricot, two subpopulations of five and six cultivars, respectively, were observed, while seven accessions were admixed. Five accessions were in common with the larger work of Bourguiba et al. (2012), one of them defining the “Adaptive Diversity” group, and three the “North Mediterranean basin” one. The fifth accession, which in their results belonged to the North Mediterranenan basin, remained admixed in our work.

Due to the lack of information, comparison with previous results was not possible in Japanese plum (K = 2). In the case of almond we could not identify a stable value of stratification. Delplancke et al. (2013) in their extensive work carried out an analysis of over 1,000 accessions, identified six clusters, but we could not make any comparison for the unavailability of common material.

Some unexpected variants, insertions or deletions, have to be inferred due to allele size differences from what could be expected from the core repeat profiles. We found both, one and two bases variants, confirmed by a second DNA extraction and analysis of all the samples where such differences were found, thus excluding PCR or electrophoresis artifacts. The tetra-nucleotide microsatellites were found more prone to include variants of two bases. This kind of variation was recorded in at least one species, in all our tetra-nucleotide SSRs with the only exception of the primer pair RPPG4-077 where the assigned allele length variations were consistent with the repetitive motif length. A similar behavior was reported in olive where some hexa-nucleotide microsatellites showed 3–5 bp differences (De la Rosa et al. 2013). The presence of variations that deviate from the core repeat multiples could be caused by complex mutation patterns as reported for *Coffea* (Poncet et al. 2006).

**Figure 3:**
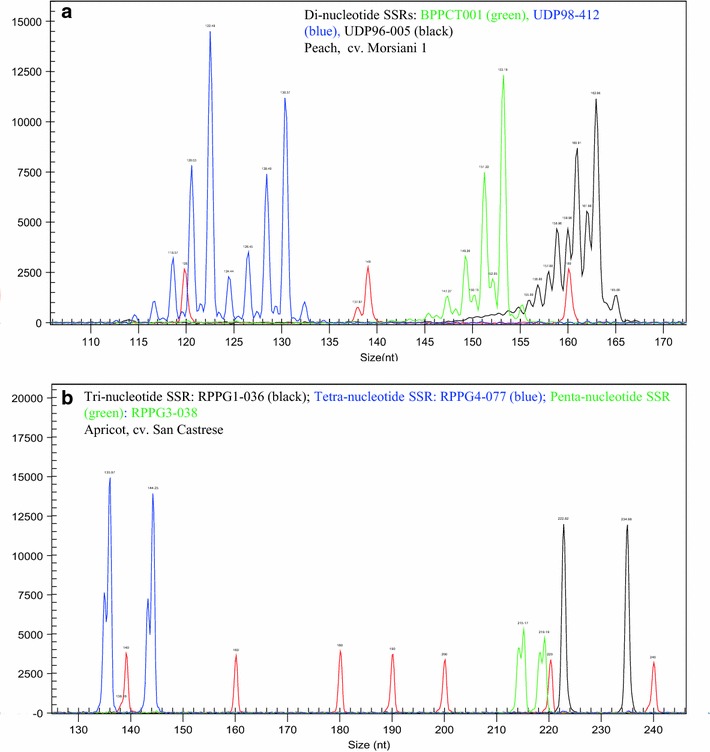
Examples of di- tri- four and penta-nucleotide SSR profiles. In red the DNA size Standard 400. **a** Profile of three di-nucleotide SSRs often used in *Prunus* analyses, each labeled with a different fluorochrome. **b** Profile of newly developed tri- tetra- and penta-nucleotide SSRs, each labeled with a different fluorochrome.

The main difference between di-nucleotide and long-core repeat microsatellite markers consist of the higher number of alleles usually displayed by the first ones, with a frequent 2 bp allelic incremental step, which results in peaks of true alleles overlapping stuttering peaks of the closest alleles (Cipriani et al. 2008). Microsatellites with longer core motifs have a lower number of alleles, larger peak distances, and stuttering peaks are attenuated, which all contribute to a more reliable scoring of microsatellites. An example of the difference among di-nucleotides and three, four- and five-nucleotides is reported in Figure 3.

## Conclusions

Access to the whole genome sequence of plants offers the opportunity to develop molecular markers tailored to different needs and purposes. Though less abundant than single nucleotide polymorphic markers (SNP), microsatellites are more efficient in low- to medium-throughput analyses where their multi-allelic nature outperforms the bi-allelic power of discrimination of SNPs. Long-core repeat microsatellites represent an advancement in the exploitation of SSR markers in fingerprinting analyses as they enable to overcome some ambiguities due to technical intrinsic issues, such as stuttering and difficulties in binning and sizing of alleles.

In this work the availability of the peach genome sequence enabled the recovery of thousands of perfect microsatellite markers with long-core repeats, namely penta-, tetra- and tri-nucleotides. A set of 26 long-core repeat markers was developed to be used in five *Prunus* species of preeminent economic importance and its effectiveness for many different purposes such as individual identification, parentage and population structure analysis was assessed. Further 190 markers were developed and tested for polymorphism in the five species and, even if they were not included in the *Prunus* set, they could still be useful for several genetic analyses.

The use of the set developed in the present work is particularly suited for all those applications where comparisons are to be made among results from different laboratories, different protocols or instruments and where molecular data have to be assigned as a reference, as is the case of database establishment or in germplasm collection management and maintenance. The straightforward scorability of these long-core SSR patterns should also simplify the task of developing multiplex PCR systems in *Prunus,* greatly improving the efficiency of genotyping. We propose to add the long-core repeat microsatellites presented here in the protocols of the future studies of individual identification of the five species of *Prunus* considered in this work.

## Additional file

Additional file 1:
**Table S1.**List of 222 primers designed and tested in a panel of 24 accessions.
